# Behavioral coping phenotypes and associated psychosocial outcomes of pregnant and postpartum women during the COVID-19 pandemic

**DOI:** 10.1038/s41598-022-05299-4

**Published:** 2022-01-24

**Authors:** Denise M. Werchan, Cassandra L. Hendrix, Jennifer C. Ablow, Ananda B. Amstadter, Autumn C. Austin, Vanessa Babineau, G. Anne Bogat, Leigh-Anne Cioffredi, Elisabeth Conradt, Sheila E. Crowell, Dani Dumitriu, William Fifer, Morgan R. Firestein, Wei Gao, Ian H. Gotlib, Alice M. Graham, Kimberly D. Gregory, Hanna C. Gustafsson, Kathryn L. Havens, Brittany R. Howell, Kathryn L. Humphreys, Lucy S. King, Patricia A. Kinser, Elizabeth E. Krans, Carly Lenniger, Alytia A. Levendosky, Joseph S. Lonstein, Rachel Marcus, Catherine Monk, Sara Moyer, Maria Muzik, Amy K. Nuttall, Alexandra S. Potter, Amy Salisbury, Lauren C. Shuffrey, Beth A. Smith, Lynne Smith, Elinor L. Sullivan, Judy Zhou, Moriah E. Thomason, Natalie H. Brito

**Affiliations:** 1 NYU Langone Health, New York, USA; 2 New York University, New York, USA; 3 University of Oregon, Eugene, USA; 4 Virginia Commonwealth University, Richmond, USA; 5 Columbia University Irving Medical Center, New York, USA; 6 Michigan State University, East Lansing, USA; 7 University of Vermont, Burlington, USA; 8 University of Utah, Salt Lake City, USA; 9 Cedars-Sinai Medical Center, Los Angeles, USA; 10 Stanford University, Stanford, USA; 11 Oregon Health and Sciences University, Portland, USA; 12 University of Southern California, Los Angeles, USA; 13 Department of Human Development and Family Science, Fralin Biomedical Research Institute at Virginia Tech Carilion, Virginia Tech, Blacksburg, USA; 14 Vanderbilt University, Nashville, USA; 15 University of Pittsburgh, Pittsburgh, USA; 16 University of Michigan, Ann Arbor, USA; 17 The Lundquist Institute at Harbor-UCLA, West Carson, USA

**Keywords:** Psychology, Human behaviour

## Abstract

The impact of COVID-19-related stress on perinatal women is of heightened public health concern given the established intergenerational impact of maternal stress-exposure on infants and fetuses. There is urgent need to characterize the coping styles associated with adverse psychosocial outcomes in perinatal women during the COVID-19 pandemic to help mitigate the potential for lasting sequelae on both mothers and infants. This study uses a data-driven approach to identify the patterns of behavioral coping strategies that associate with maternal psychosocial distress during the COVID-19 pandemic in a large multicenter sample of pregnant women (*N* = 2876) and postpartum women (*N* = 1536). Data was collected from 9 states across the United States from March to October 2020. Women reported behaviors they were engaging in to manage pandemic-related stress, symptoms of depression, anxiety and global psychological distress, as well as changes in energy levels, sleep quality and stress levels. Using latent profile analysis, we identified four behavioral phenotypes of coping strategies. Critically, phenotypes with high levels of passive coping strategies (increased screen time, social media, and intake of comfort foods) were associated with elevated symptoms of depression, anxiety, and global psychological distress, as well as worsening stress and energy levels, relative to other coping phenotypes. In contrast, phenotypes with high levels of active coping strategies (social support, and self-care) were associated with greater resiliency relative to other phenotypes. The identification of these widespread coping phenotypes reveals novel behavioral patterns associated with risk and resiliency to pandemic-related stress in perinatal women. These findings may contribute to early identification of women at risk for poor long-term outcomes and indicate malleable targets for interventions aimed at mitigating lasting sequelae on women and children during the COVID-19 pandemic.

## Introduction

The COVID-19 pandemic reflects a unique, chronic stressor that has wide-ranging consequences for psychosocial functioning across the globe. Emerging reports indicate that the COVID-19 pandemic is associated with heightened psychological distress in the general adult population^1,2^, with women and unpaid caregivers reporting disproportionate increases in symptoms of anxiety and depression^3^. The impact on pregnant and postpartum women is of particular concern given the established adverse effects of perinatal mood and anxiety disorders on the intrauterine and postnatal development of their offspring^4–6^. There is an urgent need to characterize the mental health outcomes of perinatal women during the COVID-19 pandemic and to identify risk and protective factors to minimize potentially harmful consequences during this global public health emergency. Leveraging a large, multicenter sample of pregnant and postpartum women in the United States (*N* = 4,412), we use a data-driven approach to (1) classify behavioral phenotypes of coping strategies that perinatal women are engaging in to manage pandemic-related stress, (2) isolate associations between coping phenotypes and demographic characteristics, and (3) identify coping phenotypes that are associated with risk and resiliency for adverse mental and physical health outcomes.

Pregnant and postpartum women are at heightened risk for mood and anxiety disorders, particularly following stressful life events^7,8^. The COVID-19 pandemic presents several stressors that may make perinatal women especially vulnerable to experiencing mood and anxiety disorders. For example, uncertainty regarding the impact of COVID-19 infection or vaccination on fetuses and infants, changes or disruptions in birth plans or postpartum care, and reduced access to childcare may create additional risk for maladaptive outcomes in perinatal women. Women are also more likely to work in professions that have increased virus exposure risk, such as healthcare and teaching in the United States^9^. School and childcare closures have also led to increases in unpaid domestic and childcare responsibilities taken on by women^9,10^, and have contributed to a disproportionate loss of employment in women^9^.

The impact of pandemic-related stressors on pregnant and postpartum women is of heightened concern given that stress is implicated in the etiology of perinatal mood and anxiety disorders, which can have intergenerational influences on their child’s development^6,11^. Anxiety and depression during pregnancy is associated with a number of detrimental outcomes, including increased risk of preterm birth, low birth weight, postpartum depression, and long-term adverse neurobehavioral outcomes in infants^6,11–13^. Importantly, prior systematic reviews indicate elevated rates of maternal mental issues following widespread disasters^14^. Importantly, this meta-analysis found that the severity of exposure was one of the strongest predictors of perinatal mental health outcomes following disasters. These findings elevate concerns about the impact of COVID-19-related stressors on mental health outcomes in pregnant and postpartum women, particularly given the chronic and widespread nature of these experiences. Indeed, since the start of the COVID-19 pandemic, studies across the globe have observed high rates of depression and anxiety in perinatal women^15–18^. Preventing or attenuating perinatal mood and anxiety disorders is essential for preventing a sequelae of intergenerational transmission and negative developmental consequences.

Insight into strategies to prevent potentially harmful short- and long-term consequences of pandemic-related stress and associated mood and anxiety disorders may be gleaned by exploring factors that are associated with risk and resiliency. For instance, emerging reports indicate that psychological flexibility and increased tolerance of uncertainty are associated with resiliency to pandemic-related stress^19,20^. Another, large-scale online survey of adults (*N* = 3,042) found that psychological resiliency was associated with lower COVID-19 related distress^21^. More generally, coping strategies used in response to stressful life events may increase vulnerability or resiliency for mood and anxiety disorders during the perinatal period^22^. For example, passive coping styles for managing stress (e.g., avoidance, denial), as well as social isolation and substance use, are associated with increased depressive symptoms during the prenatal and postpartum periods^23–25^. In contrast, more active coping styles (e.g., planning of action, support-seeking) and increased social support have been linked with lower perceived stress levels and resiliency to negative mental health outcomes^26–28^. Moreover, adaptive (e.g., positive appraisal) and maladaptive (e.g., avoidant) coping strategies also predict decreases and increases in emotional distress from the second to third trimester of pregnancy, respectively^29^. However, associations between maternal mental health and behavioral coping strategies, which are more immediately modifiable than psychological characteristics, have not been well characterized in perinatal women during the COVID-19 pandemic. Filling this empirical gap is critical for helping identify women most at risk for experiencing untreated mood and affective disorders and for informing scalable interventions to help mitigate lasting sequelae on women and their infants.

**Figure 1:**
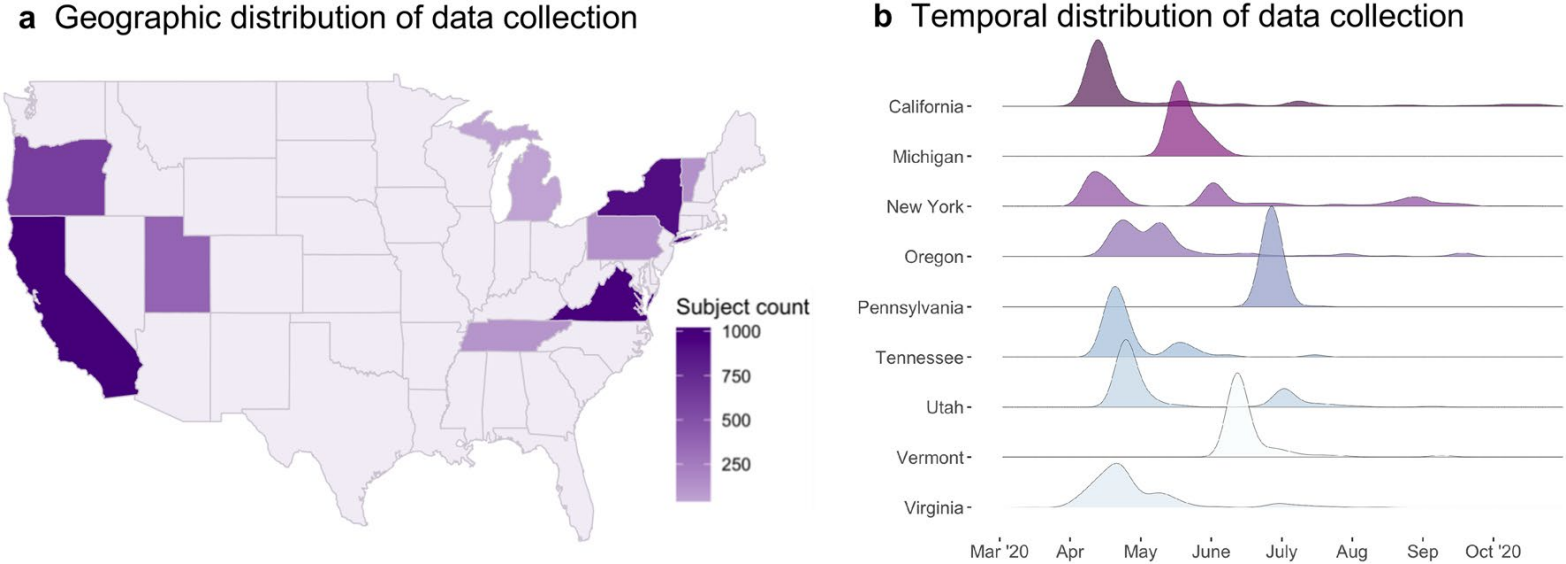
Geographic distribution and study site locations (**a**), and density plots illustrating the temporal distributions of data collection by state (**b**).

Here we use a data-driven exploratory approach to describe behavioral phenotypes of coping strategies in a large, national sample (*N* = 4,412) of pregnant and postpartum women (within the first 12 months of infant life) drawn from nine states across the United States during the first peak of the pandemic (Fig. 1). During the time this data was collected, very little was known about how the virus was spread, potential treatments, or impacts of infection on fetuses or infants. The majority of geographic regions in the United States and across the world imposed lockdown measures to contain virus spread, and the ability to get tested for COVID-19 infection was limited. Pregnant and postpartum women also experienced widespread disruptions in perinatal medical care and birth plans, which was tightly coupled with the severity of cases^30^. Thus, the time in which this data was collected reflects a particularly tumultuous period of the pandemic, with high collective uncertainty and stress. Understanding individual differences in behavioral coping patterns and associated mental health measures during this time may inform long-term impacts of early pandemic-related stress and uncertainty on women and their children.

Using this large sample of perinatal women, we first isolate behavioral coping phenotypes using latent profile analysis, which is a person-oriented analytic approach that considers how *patterns* of behaviors jointly predict outcome measures. The use of this person-oriented analytic approach allows for more precise identification of behavioral phenotypes associated with risk and resiliency, in contrast to traditional variable-oriented analytic approaches (e.g., linear regressions) that examine the contribution of individual variables to outcomes. We first identify latent dimensions of coping strategies that are reflected by the specific behaviors that women report engaging in to manage pandemic-related stress. We then use a person-oriented latent profile analysis to isolate heterogeneous subgroups of pregnant and postpartum women categorized based on similar patterns of latent coping strategies. Finally, we examine exploratory associations between profiles of coping strategies and mental and physical health outcomes. The findings from this study are essential for identifying behavioral markers of women at risk for adverse outcomes, pinpointing potential protective factors, and targeting interventions to reduce long-term consequences on women and infants.

## Results

### Descriptive statistics

**Figure 2:**
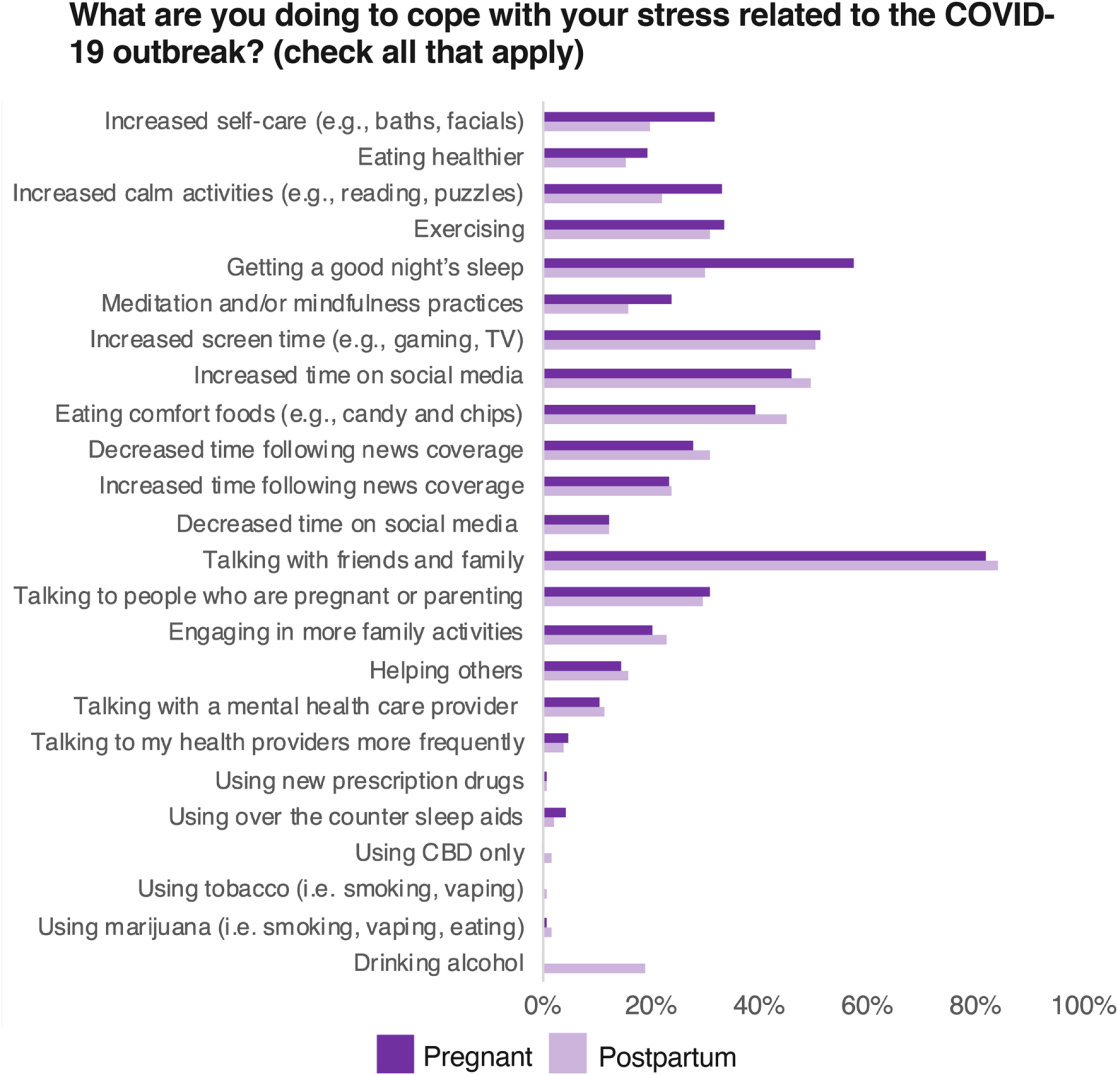
Percentage of pregnant and postpartum women endorsing each coping behavior survey item.

Demographic characteristics and means for all mental and physical health outcome variables are presented in Table 1. The proportion of participants endorsing each of the coping behavior survey items are presented in Fig. 2. As a whole, both pregnant and postpartum women reported that the COVID-19 outbreak had worsened their energy levels, sleep quality, and stress levels (all *t*s > 22.36, all *p*s < 0.001). Women also reported moderate- to high levels of COVID-related distress when asked about the overall level of stress they were experiencing due to the COVID-19 outbreak on a scale from 1 (nothing) to 7 (extreme), *M* = 4.35, *Median* = 4, *SD* = 1.5. Women also indicated symptoms of depression, anxiety and global psychological distress significantly greater than zero (all *t*s > 36.05, all *p*s < 0.001).

**Table 1:** Descriptive statistics.

Variable	Pregnant women			Postpartum women		
	N	Mean (or %)	SD	N	Mean (or %)	SD
**Demographic variables**						
Maternal race/ethnicity (% BIPOC)	2876	29%	–	1536	29%	–
Black (%)	2876	4.7%	–	1536	4.5%	–
Native American/Alaska native (%)	2876	< 1%	–	1536	< 1%	–
Native Hawaiian/Pacific Islander (%)	2876	< 1%	–	1536	< 1%	–
Asian (%)	2876	7.1%	–	1536	7.7%	–
Hispanic/Latin (%)	2876	6.7%	–	1536	5.5%	–
Two or more races/other (%)	2876	9.7%	–	1536	11.4%	–
Maternal age	2667	32.40	4.47	1419	33.15	4.57
Maternal education^a^	2824	6.91	1.41	1497	6.94	1.48
Maternal education (% 4-year college graduate)	2824	78%	–	1497	79%	–
Family income^b^	2813	8.67	4.01	1488	8.69	4.14
Number of children in the home	2796	0.79	1.10	1497	1.78	1.08
**Mental and physical health variables**						
Mean raw BSI anxiety score (0–4 range)	2859	0.65	0.73	1522	.67	0.72
Mean raw BSI depression score (0–4 range)	2859	0.80	.80	1523	.79	0.80
Mean raw BSI global score (0–4 range)	2859	.66	.64	1523	.63	.63
Change in energy levels (1 = worsened, 5 = improved)	2543	2.33	.72	1300	2.42	0.70
Change in sleep quality (1 = worsened, 5 = improved)	2843	2.56	.77	1515	2.62	0.66
Change in stress levels (1 = worsened, 5 = improved)	2728	2.11	.69	1501	2.08	.68
COVID–related distress (1 = nothing, 7 = extreme)	2734	4.30	1.50	1516	4.44	1.45

^a^Education was coded as 1 =  < 10th grade, 2 = 10–12th grade, 3 = high school/GED, 4 = apprenticeship/trade school, 5 = partial college, 6 = 2-year college, 7 = 4-year college, 8 = graduate degree.

^b^Income was coded as 1 =  < 10 k, 2 = 10–20 k, 3 = 20–30 k, 4 = 30–40 k, 5 = 40–50 k, 6 = 50–60 k, 7 = 60–80 k, 8 = 80–100 k, 9 = 100–120 k, 10 = 120–140 k, 11 = 140–160 k, 12 = 160–180 k, 13 = 180–200 k, 14 = 200–220 k, 15 = 220–250 k, 16 = 250 k + .

### Identification of latent dimensions of coping strategies

We used a principal components analysis with promax rotation, which permits correlation between the factors, to identify latent dimensions of coping strategies reflected by the original survey items (Fig. 2). Examination of the scree plot suggested a 6-factor solution, which was conceptually appropriate and accounted for 38.7% of the overall variance. One item from the original survey (“Other”) did not load on any factors above 0.2 and was thus removed. All remaining items had loadings above 0.45, with the exception of “Using CBD only”, which showed a marginally lower loading of 0.37. There were no substantial cross-loadings of variables onto multiple factors. The full structure matrix indicating factor loadings for each survey item in the final 6-factor solution are presented in Table 2.

**Table 2:** Principal components analysis structure matrix and factor correlations.

	Factor 1	Factor 2	Factor 3	Factor 4	Factor 5	Factor 6
**Survey item**						
Increased self-care (e.g., baths, facials)	0.63					
Eating healthier	0.57					
Increased calm activities (e.g., reading, puzzles)	0.55					
Exercising	0.54					
Getting a good night’s sleep	0.49					
Meditation and/or mindfulness practices	0.46					
Increased screen time (e.g., gaming, TV)		0.76				
Increased time on social media		0.73				
Eating comfort foods (e.g., candy and chips)		0.63				
Decreased time following news coverage			0.80			
Increased time following news coverage			− 0.68			
Decreased time on social media			0.60			
Talking with friends and family				0.62		
Talking to people who are pregnant or parenting				0.55		
Engaging in more family activities				0.52		
Helping others				0.47		
Talking with a mental health care provider					0.61	
Talking to my health providers more frequently					0.57	
Using new prescription drugs					0.55	
Using over the counter sleep aids					0.46	
Using CBD only						0.37
Using tobacco (i.e. smoking, vaping)						0.65
Using marijuana (i.e. smoking, vaping, eating)						0.57
Drinking alcohol						0.49
**Factor correlations**						
Factor 1 (self-care)	1					
Factor 2 (vegging out)	− 0.06	1				
Factor 3 (avoiding media/news)	0.14	− 0.13	1			
Factor 4 (social support)	0.20	0.05	0.09	1		
Factor 5 (healthcare utilization)	0.07	0.12	0.05	0.05	1	
Factor 6 (substance use)	− 0.02	0.01	− 0.03	− 0.07	− 0.07	1

*N* = 4,412. Component loadings below |.30| are suppressed for ease of presentation.

Factor 1 reflects the latent dimension of “self-care” and consists of exercising, getting a good night’s sleep, meditation, eating healthy, self-care activities, and calm activities. Factor 2 reflects “vegging out” and consists of increased screen time, social media use, and comfort foods. Factor 3 reflects “avoiding media/news” and consists of decreased social media use, decreased time following news, and increased time following news (reverse scored). Factor 4 embodies “social support” and consists of talking with friends and family, helping others, engaging in family activities, and talking to other parents/pregnant women. Factor 5 reflects “healthcare utilization” and consists of talking to healthcare providers, talking to mental health providers, using new prescription drugs, and using over-the-counter sleep aids. Factor 6 reflects “substance use” and consists of tobacco use, marijuana, CBD, and alcohol consumption. Correlations among factors ranged from − 0.13 to 0.20. Composite reliability (CR) for the identified coping strategy subscales was evaluated using McDonald’s omega. Internal consistency was adequate for self-care (CR = 0.70), vegging out (CR = 0.74), avoiding media/news (CR = 0.89), substance use (CR = 0.75), and healthcare utilization (CR = 0.72), based on recommended guidelines of composite reliability equal to or greater than 0.60 for exploratory scales^31^. Social support had relatively lower internal consistency (CR = 0.55), but we retained this composite given the conceptual fit and high factor loadings of the items in this construct.

Composite variables representing the latent coping strategies were created by collapsing across the original survey items. Specifically, composite variables for “self-care”, “social support”, “avoiding media/news”, and “vegging out” were created by averaging over the individual items reflecting each of these constructs. Composite variables for “substance use” and “healthcare utilization” were created by discretizing the individual items into a binary categorical variable (none, or 1 + items). We did this to prevent floor effects resulting from the low proportion of responses across these items (see Fig. 2), which would have prevented model convergence in subsequent latent profile analyses. We created composite variables by collapsing over variables, rather than using the latent factor scores, as this method retains the variance in the original data and is superior when using exploratory scales^32^.

### Identification of maternal behavioral coping phenotypes

**Figure 3:**
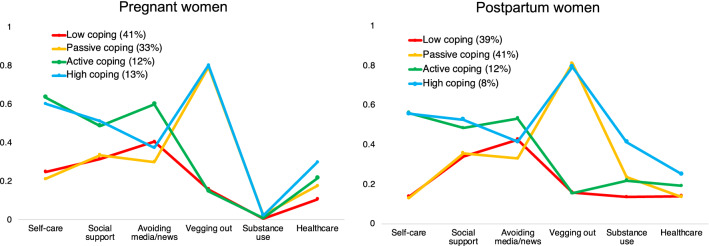
Estimated means for the 6 coping strategies across all profiles for both pregnant women and postpartum women. Differing levels of vegging out, self-care, and social support were key factors that most strongly differentiated profiles in both pregnant and postpartum women.

We examined four models, comparing the fit of 2–5 possible profiles. We stopped once the LMR test became non-significant, thus indicating that a simpler model with one less profile was a better fit of the data. Separate models were fit for pregnant and postpartum women using the composite coping strategy variables as indicators. The 4-profile model was the best fitting model for both pregnant and postpartum women, as reflected by a combined low BIC, a significant LMR test, a high entropy value, and each profile representing at least 5% of the entire sample (see *Supplementary Information* Table S1 for full results). The latent structures of the profiles are presented in Fig. 3, which illustrate mean values for the continuous and categorical coping strategy variables for each profile. Vegging out, self-care, and social support were key factors that most strongly differentiated profiles (*Supplementary Information,* Table S2 and Table S3). Similar profiles were found for pregnant and postpartum women and are described below.

**Profile 1—Low-coping.** The first profile extracted accounted for 41% of the sample in pregnant women (n = 1188), and 39% of the sample in postpartum women (n = 595). This profile was characterized by moderate endorsement of avoiding media/news, and relatively lower endorsements of all other coping strategies.

**Profile 2—Passive-coping.** The second profile extracted accounted for 33% of the sample in pregnant women (n = 960), and 41% of the sample in postpartum women (n = 635). This profile was characterized by high levels of vegging out, and lower levels of self-care and social support.

**Profile 3—Active-coping.** The third profile extracted accounted for 12% of the sample in both pregnant women (n = 349) and in postpartum women (n = 178). This profile was characterized by endorsing high levels of self-care, social support, and avoiding media/news, and low levels of vegging out.

**Profile 4—High-coping.** The final profile extracted accounted for 13% of the sample in pregnant women (n = 379), and 8% of the sample in postpartum women (n = 128). This profile was characterized by high levels of self-care, social support, and vegging out. This profile was also characterized by moderately increased healthcare utilization, as well as moderately elevated substance use in the postpartum women only.

### Demographic and socioeconomic determinants of coping phenotypes

We examined demographic and socioeconomic predictors of profile membership using the 3-step procedure for predictor variables (“R3STEP” command in Mplus; see *Supplementary Information,* Table S4 and Table S5 for full results). Relative to women in the low-coping profile, pregnant women in the active-coping profile were more likely to have fewer children (odds ratio [OR] = 0.64, 95% CI 0.52–0.78), and were less likely to identify as Black (OR = 0.30, 95% CI 0.12–0.73) or Asian (OR = 0.42, 95% CI 0.21–0.84). Pregnant women in the high-coping profile were also more likely to have fewer children (OR = 0.61, 95% CI 0.43–0.87) and greater educational attainment (OR = 1.15, 95% CI 1.03–1.28). Pregnant women in the passive-coping profile were marginally more likely to have greater educational attainment (OR = 1.004, 95% CI 1.002–1.006), and were less likely to identify as Black (OR = 0.65, 95% CI 0.46–0.92).

Relative to the low-coping profile, postpartum women in the passive-coping profile were more likely to have greater educational attainment (OR = 1.13, 95% CI 1.04–1.23), to be younger in age (OR = 0.96, 95% CI 0.94–0.99), and to identify as Black (OR = 2.33, 95% CI 1.42–3.84). Postpartum women in the active-coping and high-coping coping profiles were more likely to have greater educational attainment (active-coping: OR = 1.26, 95% CI 1.07–1.48; high-coping: OR = 1.25, 95% CI 1.07–1.47). No other variables were significant predictors of profile membership in postpartum women.

### Associations with mental and physical health outcomes

A critical aim was to determine whether profile membership predicted differences in mental and physical health outcomes to identify potential risk and protective factors. To examine this, we used the 3-step auxiliary approach for distal outcomes with unequal means and equal variances (“DE3STEP” command in Mplus). This approach accounts for measurement error associated with most likely profile membership and is shown to be superior to other methods^33^.

**Figure 4:**
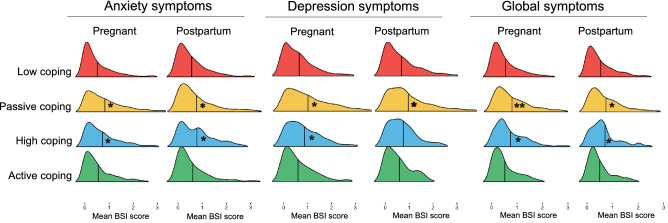
Distributions of anxiety, depression, and global BSI scores by profile for pregnant and postpartum women, based on most likely profile membership. Solid lines indicate estimated means. Asterisks indicate which profiles had significantly higher means.

In pregnant women, membership in a particular profile predicted differences in all outcome measures (Table 3). Women with coping profiles characterized by high levels of vegging out (high-coping and passive-coping) were more likely to have greater symptoms of anxiety, depression and global psychological distress relative to other profiles (Fig. 4). Women in these profiles also reported worsening stress levels, and overall greater COVID-related distress relative to women in the active-coping and low-coping profiles. Women with profiles characterized by high self-care and social support (active-coping and high-coping) also reported fewer negative changes in sleep quality as a result of the COVID-19 outbreak. Additionally, women in the active-coping profile reported fewer changes in energy levels relative to the other profiles.

**Table 3:** Comparisons of mental and physical outcome measures between profiles for both pregnant and postpartum women.

	Active-coping	High-coping	Passive-coping	Low-coping	Omnibus χ^2^ test
	M (SE)	M (SE)	M (SE)	M (SE)	
**Pregnant women**					
BSI anxiety	.56 (.04)^a^	.70 (.05)^b^	.85 (.03)^b^	.50 (.02)^a^	χ2(3) = 101.6, *p* < .001
BSI depression	.60 (.04)^a^	.81 (.05)^b^	1.05 (.04)^b^	.64 (.04)^a^	χ2(3) = 133.8, *p* < .001
BSI global	.52 (.03)^a^	.72 (.04)^b^	.83 (.03)^c^	.55 (.02)^a^	χ2(3) = 103.7, *p* < .001
Change in energy	2.56 (.05)^a^	2.33 (.05)^b^	2.16 (.03)^c^	2.39 (.02)^b^	χ2(3) = 70.97, *p* < .001
Change in sleep	2.79 (.05)^a^	2.68 (.06)^a,b^	2.34 (.03)^c^	2.59 (.02)^b^	χ2(3) = 61.53, *p* < .001
Change in stress	2.29 (.05)^a^	2.06 (.05)^b^	1.96 (.05)^b^	2.20 (.02)^a^	χ2(3) = 82.32, *p* < .001
COVID-related distress	4.21 (.11)^a^	4.49 (.08)^b^	4.63 (.05)^b^	4.00 (.05)^a^	χ2(3) = 90.03, *p* < .001
**Postpartum women**					
BSI anxiety	.60 (.06)^a,b^	.79 (.08)^b^	.76 (.03)^b^	.56 (.03)^a^	χ2(3) = 28.38, p < .001
BSI depression	.59 (.06)^a^	.74 (.06)^a^	.98 (.04)^b^	.67 (.03)^a^	χ2(3) = 53.54, p < .001
BSI global	.50 (.04)^a^	.70 (.07)^b^	.74 (.03)^b^	.54 (.03)^a^	χ2(3) = 41.05, p < .001
Change in energy	2.62 (.06)^a^	2.44 (.07)^a,b^	2.32 (.03)^b^	2.45 (.03)^a^	χ2(3) = 21.73, p < .001
Change in sleep	2.69 (.05)^a^	2.66 (.07)^a^	2.57 (.03)^a^	2.63 (.03)^a^	χ2(3) = 5.35, p = .15
Change in stress	2.27 (.06)^a^	2.17 (.07)^a^	1.98 (.03)^b^	2.12 (.03)^a^	χ2(3) = 26.50, p < .001
COVID-related distress	4.24 (.13)^a^	4.47 (.14)^a,b^	4.68 (.06)^b^	4.25 (.07)^a^	χ2(3) = 26.27, p < .001

Superscripts indicate which groups differ based on significant (*a* < .05) Wald tests, with Holm-Bonferroni corrections for multiple comparisons.

In postpartum women, latent profile membership predicted all outcome variables with the exception of changes in sleep quality (Table 3). Of note, women in the passive-coping profile reported elevated depression symptoms (Fig. 4), and worsening stress levels relative to other women. Women with profiles characterized by high levels of vegging out (high-coping and passive-coping) also reported increased anxiety and global psychological distress symptoms (Fig. 4), and moderately worsening energy levels relative to other profiles.

## Discussion

In addition to presenting a number of unique stressors, COVID-19 has disrupted routines, support systems, and behavioral strategies that previously may have been beneficial for managing stress. The impact of pandemic-related stress on perinatal women is of heightened concern, given the established intergenerational transmission of maternal stress-exposure^5,6^, and the disproportionate burdens experienced by women during the COVID-19 pandemic. Identifying behavioral phenotypes that are related to risk and resiliency for negative outcomes is essential for targeting resources and interventions for those most vulnerable. To address this urgent need, we use a data driven approach to identify four distinct behavioral phenotypes of adaptive and maladaptive coping strategies in a large, national sample of perinatal women in the United States (Fig. 3).

We found that engaging in passive coping strategies (increased screen time, social media, and intake of comfort foods), social support, and self-care were key factors differentiating these behavioral coping phenotypes, and that profile membership also differed by sociodemographic characteristics. Relative to the low-coping reference profile, pregnant women in the active-coping and high-coping profiles were more likely to have fewer children. In addition, greater educational attainment was associated with high-coping and passive-coping in pregnant women, as well as high-coping and active-coping in postpartum women. When considering race and ethnicity, we found that pregnant women in the active-coping and passive-coping profiles were less likely to identify as Black, and those in the active coping profile were less likely to identify as Asian. These findings demonstrate that the probability of belonging in a particular coping phenotype varies by race/ethnicity and sociodemographic characteristics of our sample, which is noteworthy given differential associations between profile membership and mental and physical health variables. However, these findings should be interpreted with caution given that our sample was skewed towards higher SES and White participants (see Table 1).

When examining measures of physical and mental health, as a whole, both pregnant and postpartum women reported worsening changes in sleep quality, energy levels, and stress levels relative to pre-pandemic levels. These results are consistent with emerging reports indicating worsening psychosocial functioning in both pregnant women and in the general adult population across the globe^1–3,18,21,34^. We add novel insights into potential behavioral risk and protective factors for adverse psychosocial functioning by showing that these associations differ based on behavioral coping phenotypes. Women who reported high levels of vegging out (the high-coping and passive-coping profiles) also reported the largest negative change in energy and stress levels, as well as the highest levels of global psychological distress scores relative to other profiles. In contrast, women who primarily reported engaging in active-coping behaviors also reported fewer negative changes in sleep quality and energy levels relative to other women, particularly among pregnant women. Similarly, women who reported lower levels of all coping behaviors also reported fewer negative changes in mental and physical health outcomes, as well as lower levels of distress related to the COVID-19 pandemic.

Importantly, in our sample we observed that symptoms relevant to anxiety and depression differed based on coping phenotype. Both pregnant and postpartum women in the passive-coping and high-coping phenotypes reported greater symptoms of both anxiety and depression relative to women in the active-coping and low-coping profiles (Fig. 4). Postpartum women who supplemented passive-coping with more active strategies (high-coping) exhibited fewer depression symptoms relative to women who only engaged in passive strategies (passive-coping). In contrast, in both pregnant and postpartum, supplementing passive coping strategies with active coping strategies was not associated with fewer anxiety symptoms relative to women who only engaged in passive strategies. While we cannot estimate prevalence of probable clinical diagnoses of depression or anxiety in our sample, given that the BSI-18 is not a standardized measure for clinical evaluations during the perinatal period, these findings suggest robust associations between behavioral coping phenotypes and symptoms of depression and anxiety during the perinatal period. Our results are largely consistent with recent evaluations of perinatal women during the COVID-19 pandemic, which suggest stark increases in probable clinical depression and anxiety. Indeed, a recent meta-analysis of mental health in pregnant women during the COVID-19 pandemic across the glove estimated that worldwide prevalence rates of depression and anxiety to be 25.6% and 30.5%, respectively, with few differences based on geographic region, maternal characteristics, or timing of data collection^35^. This is contrast to pre-pandemic meta-analyses, which estimated prevalence rates of perinatal anxiety and depression in the United States to be approximately 20.7% and 11.9%, respectively^36,37^.

Our findings also align with clinical models of depression, which emphasize the role of inactivity as both a symptom of and contributor to depression^38^. Our findings also align with recent reports indicating that higher levels of physical activity are associated with less COVID-19-related stress in pregnant women across the globe^18,34,39^. However, we cannot ascertain that our findings are directly tied to the COVID-19 pandemic, given the lack of a matched pre-pandemic comparison group. Indeed, these behavioral phenotypes may not be unique to the COVID-19 pandemic but may reflect more generalizable patterns of coping strategies in response to stressful life events. For instance, prior work has similarly observed that engaging in passive psychological coping styles, such as avoidance or denial, is associated with depression symptoms during the prenatal and postpartum periods^23–25,40^. Maladaptive coping is also predictive of increases in emotional distress from mid- to late pregnancy in pre-pandemic samples^29^. Yet, it is noteworthy that in our sample of women recruited during the first peak of the COVID-19 pandemic, we observed a concerningly large percentage of both pregnant and postpartum women in the passive-coping behavioral phenotype (33% and 41%, respectively).

In pregnant women, supplementing high levels of passive coping strategies with active strategies (*i.e.,* the high-coping profile) was not associated with fewer depression symptoms relative to women who only endorsed passive strategies. This result is in contrast to our findings in postpartum women, where we observed fewer depression symptoms among women in the high-coping profile relative to those in the passive-coping profile. These differences could relate to pregnant versus postpartum women’s motivation for engaging in active coping strategies. For instance, pregnant women may engage in active strategies such as increased physical activity and healthy eating for the wellbeing of their unborn baby, whereas postpartum women may be more likely to engage in these activities for their own health and wellbeing. Nonetheless, these findings indicate that “vegging out” may be an important behavioral marker for adverse outcomes, particularly in pregnant women. This knowledge can be used to target interventions, possibly even prior to the emergence of clinically elevated symptoms. For instance, clinicians could use wearable activity monitors or ask questions probing physical activity and nutrition to help identify and target interventions for women at risk for adverse outcomes. Clinicians could additionally use information regarding engagement in positive coping strategies, such as social support and self-care activities, in determining potential protective factors when evaluating risk for perinatal mood disorders. In addition, future clinical assessments could evaluate the efficacy of interventions aimed at treating mood disorders through increasing activity, such as behavioral activation therapy, in peripartum women at risk for poor outcomes. This information may be especially relevant given that a number of women often do not report symptoms or seek treatment for perinatal mood and anxiety disorders, in part due to stigma or fear of teratogenic effects with medication use^41^.

These findings should be interpreted within the limitations of our data. First, we lack evidence of prior psychiatric diagnoses, as well as data on the prevalence of COVID-19 infection in our sample, given that the majority of data were collected at the start of the pandemic when PCR or antigen tests were difficult to obtain. It is possible that some differences in coping strategies, and associated depressive and anxiety symptoms, could be due to COVID-19 infection. Indeed, increasing evidence suggests that COVID-19 infection may increase risk of developing mood disorders^42^. This is an important topic to consider in future work. In addition, our sample was relatively homogenous, with the majority of subjects identifying as White and from higher income households. Moreover, these findings are only representative of perinatal women in the United States, and future work is needed to assess the generalizability of these patterns across different geographic regions and cultures. Importantly, our findings also reflect a data-driven descriptive analysis of behavioral coping phenotypes and their associations with symptoms of depression and anxiety, rather than an experimental or longitudinal investigation of these questions. Thus, we cannot ascertain whether these findings are specific to the pandemic or whether women’s depression and anxiety symptoms were higher than what they may have experienced prior to the pandemic. As such, these correlational findings should not be used to infer causal assumptions, but rather should be used to inform future hypotheses and experimental studies examining associations between behavioral coping strategies and physical and mental health during the perinatal period.

In sum, this large-scale, multicenter study highlights the impact of the COVID-19 pandemic on mental and physical health outcomes of perinatal women in the United States. It identifies widespread patterns of coping strategies that perinatal women are engaging in to manage pandemic-related stress, with differing associations with mental and physical health outcomes. The behavioral coping phenotypes we identified highlight potential risk and protective factors for perinatal women, which is critical in helping to identify and treat women most at risk for experiencing mood and anxiety disorders during the COVID-19 pandemic and other global health crises.

## Method

### Participants and procedures

Participants were recruited into studies examining the impact of COVID-19 on perinatal women taking place at 14 academic research institutions across nine states (Fig. 1a). Criteria for participation included being pregnant or postpartum within the first 12 months of infant life. Women were recruited using medical records or through existing participant pools, depending on site of recruitment. The study was approved by the Institutional Review Board at NYU Langone (study title “COPE Study: COVID-19 and Perinatal Experiences”, study number: i20-00383) and at each local site. All methods were carried out in accordance with relevant guidelines and regulations. The studies were independent, but the investigators used common research methods to facilitate cross-site data sharing. All sites administered the COPE: COVID-19 & Perinatal Experiences—Impact Survey^43^ online between March and October 2020, with the majority of data collection occurring in April 2020 (Fig. 1b). Informed electronic consent was obtained prior to data collection. The combined sample consisted of 4,412 women (2,876 pregnant women, 1,536 postpartum women) drawn from 9 states (Fig. 1a). Full demographic characteristics of the final sample and means for all outcome variables are presented in Table 1. The survey was administered to an additional 1,294 women, but their data were excluded due to failure to complete the “Adjustment and Coping” subsection of the survey (n = 814), or due to inconsistent or unreliable survey response patterns (n = 37 due to selecting both “increased” and “decreased” behaviors; n = 389 due to selecting “None” in addition to other behaviors). We additionally excluded n = 54 women who reported no coping behaviors.

### Measures

#### Coping behaviors

Women were asked “What are you doing to cope with your stress related to the COVID-19 outbreak? (check all that apply).” The specific survey items and proportion of subjects endorsing each item are listed in Fig. 2. The survey items probed a broad spectrum of adaptive and maladaptive behaviors, including social support mechanisms, physical activity, nutrition, substance use, healthcare utilization, news consumption, screen time, and self-care related activities (Fig. 2). One additional item (“using other recreational drugs”) in the original survey was removed for analyses due to fewer than 5 responses to this question across participants. The survey items were selected by the research team during the initial rapid design of the COPE: COVID-19 & Perinatal Experiences—Impact Survey in March 2020. The coping items were selected to encompass a range of physical, emotional, and social support mechanisms that were accessible during the pandemic and that were hypothesized to have predictive value for risk and resiliency for poor mental and physical health outcomes.

#### Mental and physical health outcomes

We collected data on how the COVID-19 pandemic changed women’s sleep, energy levels, and overall stress by asking women to report “How has the COVID-19 outbreak changed your stress levels or mental health/sleep/daily energy levels?” using 5-point Likert scales (1 = worsened significantly, 2 = worsened moderately, 3 = no change, 4 = improved moderately, 5 = improved significantly). We also collected data on women’s overall COVID-19 related distress by asking women to report their “Overall level of stress related to the COVID-19 outbreak” using a 7-point Likert scale (1 = nothing, 7 = extreme). Anxiety, depression and global psychological distress symptoms were measured using the Brief Symptom Inventory (BSI-18), which is a standardized questionnaire of psychological symptoms^44^. The BSI asks subjects to indicate how much distress they have experienced from each symptom in the past two weeks on a 5-point Likert-type scale (0 = not at all; 4 = extremely). The suicidality item was omitted in survey administration, leaving five items probing depressive symptoms, six probing anxiety symptoms, and six probing somatic symptoms. Depression and anxiety symptom scores were calculated by averaging across each subscale, and global BSI scores were calculated by averaging across all 17 items. Reliability was high for the overall scale (Chronbach’s alpha = 0.894, 95% CI [0.89, 0.90]), as well as for the depression subscale (Chronbach’s alpha = 0.81, 95% CI [0.80, 0.82]) and the anxiety subscale (Chronbach’s alpha = 0.81, 95% CI [0.80, 0.814]).

### Analytic plan

We first conducted an exploratory principal components analysis to reduce the survey items into composite variables representing different dimensions of coping strategies using SPSS version 21.0. We then used latent profile analysis in Mplus Version 8.1 to identify groups of pregnant and postpartum women categorized based on similar patterns of coping strategies. Separate models were fit for pregnant women and postpartum women to account for potential differences in patterns of coping strategies. Each model was initialized 200 times, with 50 iterations for the final stage of optimization. Using established guidelines for model selection, the best fitting model was determined by: a low Bayesian information criterion (BIC), a high entropy value (indicating low classification error), at least 5% of the total participant count in a given profile, and a significant Lo–Mendell–Rubin likelihood ratio test (LMR)^45^. Note that a significant LMR test does not identify good model fit alone, but rather suggests that a model with one less profile is a better fit of the data. Thus, LPA models were fit until the LMR test became non-significant.

We examined demographic and socioeconomic variables that were predictive of latent profile membership using the 3-step procedure for predictor variables (“R3STEP” command for multinomial logistic regression in Mplus). Finally, we used the 3-step auxiliary approach for outcome measures with unequal means and equal variances (“DE3STEP” command in Mplus) when examining associations between profile membership and distal outcomes (stress levels, sleep quality, energy levels, symptoms of depression and anxiety, and overall COVID-related distress). This approach accounts for measurement error associated with most likely profile membership and is shown to be superior to other methods^33^. However, note that these analyses cannot make causal assumptions about predictors or outcomes, given that all data were collected cross-sectionally.

## Data Availability

The datasets generated during and/or analyzed during the current study are available from the corresponding author on reasonable request.

## Supple mentary Information

Suppl ementary In formation.
