# Supplementary material for: Behavioral coping phenotypes and associated psychosocial outcomes of pregnant and postpartum women during the COVID-19 pandemic
Source: Sci Rep. 2022 Jan 24;12:1209. doi: 10.1038/s41598-022-05299-4 (PMC8786860; doi:10.1038/s41598-022-05299-4)
Supplement: Supplementary file 1 — Suppl ementary In formation. [file 41598_2022_5299_MOESM1_ESM.pdf]

Supplementary Information

Behavioral coping phenotypes and associated psychosocial outcomes of pregnant and postpartum women during the COVID-19 pandemic

Denise M. Werchan[1], Cassandra Hendrix[1], Jennifer C. Ablow[3], Ananda B. Amstadter[4], Autumn C. Austin[1], Vanessa Babineau[5], G. Anne Bogat[6], Leigh-Anne Cioffredi[7], Elisabeth Conradt[8], Sheila Crowell[8], Dani Dumitriu[5], William Fifer[5], Morgan Firestein[5], Wei Gao[9], Ian H. Gotlib[10], Alice Graham[11], Kimberly D. Gregory[9], Hanna C. Gustafsson[11], Kathryn L. Havens[12], Brittany R Howell[13], Kathryn L. Humphreys[14], Lucy S. King[10], Patricia Kinser[4], Elizabeth E. Krans[15], Carly Lenniger[1], Alytia A. Levendosky[6], Joseph S. Lonstein[6], Rachel Marcus[9], Catherine Monk[5], Sara Moyer[4], Maria Muzik[16], Amy K. Nuttall[6], Alexandra S Potter[7], Amy Salisbury[4], Lauren C. Shuffrey[5], Beth A. Smith[12], Lynne Smith[17], Elinor L. Sullivan[11], Judy Zhou[12], Moriah E. Thomason[1]*, & Natalie H. Brito[2]*

Denise M Werchan
Email: denise.werchan@nyulangone.org

Table S1. LPA model fits for pregnant and postpartum women.

	**BIC**		**Entropy**		**Smallest profile (%)**		**LMR *p* value**	
	Pregnant	*Postpartum*	*Pregnant*	*Postpartum*	*Pregnant*	*Postpartum*	*Pregnant*	*Postpartum*
2-profile	5521	4142	.90	.90	47%	50%	< .001	< .001
3-profile	5352	3979	.82	.85	7%	7%	< .001	.04
4-profile	5155	3810	.78	.86	12%	8%	< .001	< .001
5-profile	5166	3793	.76	.84	6%	2%	.41	.75

Note. BIC = Bayesian Information Criteria; LMR = Lo-Mendell-Rubin.

Table S2. Mean comparisons of coping strategies between profiles for pregnant women.

	Low-coping (n = 1188)	Passive-coping (n = 960)	Active-coping (n = 349)	High-coping (n = 379)		
	M (SD)	*M (SD)*	*M (SD)*	*M (SD)*	*F*	η_p^2
**Self-Care**	.24 (.17) ^{b}	.20 (.15) ^{b}	.67 (.15) ^{a}	.63 (.16) ^{a}	1298.72	.58
**Social Support**	.31 (.21) ^{b}	.33 (.21) ^{b}	.50 (.23) ^{a}	.53 (.23) ^{a}	156.65	.14
**Avoiding media/news**	.40 (.26) ^{b}	.30 (.26) ^{c}	.61 (.30) ^{a}	.37 (.30) ^{b}	109.71	.10
**Vegging out**	.16 (.17) ^{b}	.80 (.16) ^{a}	.15 (.17) ^{b}	.81 (.17) ^{a}	3706.66	.80
**Substance Use**	.01 (.09) ^{b}	.02 (.15) ^{a}	.01 (.09) ^{a}	.02 (.14) ^{a}	3.50	<.01
**Healthcare**	.11 (.31) ^{c}	.17 (.11) ^{b}	.23 (.42) ^{b}	.31 (.46) ^{a}	32.07	.03

Differing subscripts within rows indicate significantly different means at $p < .05$, with Tukey's HSD correction.

Table S3. Mean comparisons of coping strategies between profiles for postpartum women.

	**Low-coping (n = 595)**	**Passive-coping (n = 635)**	**Active-coping (n = 178)**	**High-coping (n = 128)**		
	M (SD)	*M (SD)*	*M (SD)*	*M (SD)*	*F*	η_p^2
**Self-Care**	.14 (.12) ^{b}	.13 (.13) ^{b}	.59 (.14) ^{a}	.60 (.14) ^{a}	1017.26	.67
**Social Support**	.34 (.21) ^{b}	.36 (.22) ^{b}	.47 (.26) ^{a}	.52 (.25) ^{a}	30.27	.06
**Avoiding media/news**	.43 (.29) ^{b}	.34 (.27) ^{c}	.52 (.33) ^{a}	.41 (.34) ^{b}	22.70	.04
**Vegging out**	.16 (.17) ^{b}	.82 (.17) ^{a}	.16 (.17) ^{b}	.81 (.17) ^{a}	2019.52	.80
**Substance Use**	.14 (.34) ^{c}	.24 (.43) ^{b}	.24 (.43) ^{b}	.41 (.50) ^{a}	18.81	.04
**Healthcare**	.14 (.35) ^{b}	.14 (.35) ^{b}	.19 (.39) ^{b}	.26 (.44) ^{a}	4.53	.01

Differing subscripts within rows indicate significantly different means at $p < .05$, with Tukey's HSD correction.

Table S4. Multinomial logistic regression of predictors of latent profile membership for pregnant women.

	High-coping (vs. low)			Passive-coping (vs. low)			Active-coping (vs. low)		
	B	*SE*	*Odds ratio*	*B*	*SE*	*Odds ratio*	*B*	*SE*	*Odds ratio*
**Black** [a]	-0.62	.41	0.54	-0.43*	0.21	0.65	-1.21*	0.55	0.30
**Hispanic/Latin** [a]	-0.46	.32	0.63	-0.32	0.17	0.73	-0.44	0.31	0.65
**Asian** [a]	-0.10	0.26	0.90	-0.10	0.18	0.91	-0.88*	0.42	0.42
**Two or more races/other** [a]	-0.16	0.38	0.85	0.13	0.22	1.14	0.48	0.14	1.61
**Maternal age**	-0.02	0.02	0.98	-0.02	0.01	0.98	0.03	0.02	1.03
**Children**	-0.49*	0.22	0.61	-0.07	0.05	0.92	-0.46**	0.13	0.64
**Education**	0.14*	0.07	1.15	0.004*	0.001	1.004	0.00	0.00	1.00
**Income**	0.00	0.02	1.01	0.01	0.02	1.01	-0.04	0.03	0.96

[a] Race/ethnicity was dummy-coded, with white as the reference category
* $p < .05$
** $p < .01$

Table S5. Multinomial logistic regression of predictors of latent profile membership for postpartum women.

	High-coping (vs. low)			Passive-coping (vs. low)			Active-coping (vs. low)		
	B	*SE*	*Odds ratio*	*B*	*SE*	*Odds ratio*	*B*	*SE*	*Odds ratio*
**Black** [a]	0.18	0.62	1.20	0.85**	0.30	2.33	0.70	0.46	2.01
**Hispanic/Latin** [a]	-0.63	0.47	0.54	0.11	0.22	1.11	-0.20	0.38	0.82
**Asian** [a]	-0.40	0.47	0.67	-0.10	0.24	0.90	-0.35	0.45	0.70
**Two or more races/other** [a]	0.55	0.48	1.73	0.18	0.33	1.20	0.45	0.46	1.56
**Maternal age**	-0.04	0.03	0.96	-0.04*	0.02	0.96	-0.05	0.03	0.95
**Children**	-0.22	0.27	0.80	0.02	0.07	1.02	-0.12	0.10	0.89
**Education**	0.23*	0.10	1.25	0.12*	0.05	1.13	0.23*	0.10	1.26
**Income**	-0.04	0.03	1.20	0.02	0.02	1.02	0.00	0.03	1.00

[a] Race/ethnicity was dummy-coded, with white as the reference category
* $p < .05$
** $p < .01$
